# Pheromonal Communication in the European House Dust Mite, *Dermatophagoides pteronyssinus*

**DOI:** 10.3390/insects5030639

**Published:** 2014-08-08

**Authors:** Johannes L.M. Steidle, Elena Barcari, Marc Hradecky, Simone Trefz, Till Tolasch, Cornelia Gantert, Stefan Schulz

**Affiliations:** 1 Institut für Zoologie, Fachgebiet Tierökologie 220c, Universität Hohenheim, Garbenstr. 30, Stuttgart 70593, Germany; E-Mails: jsteidle@uni-hohenheim.de (J.L.M.S.); tolasch@uni-hohenheim.de (T.T.); cgantert@uni-hohenheim.de (C.G.); 2 Institut für Organische Chemie, Technische Universität Braunschweig, Hagenring 30, Braunschweig 38106, Germany; E-Mail: elenabarcari@yahoo.com; 3 Castello fine arts GmbH, Fachbereich Material und Umweltanalytik, Rechberghäuser Weg 11 Göppingen, Baden-Württemberg 73035, Germany; E-Mail: marc.hradecky@cfa-analytics.de; 4 Pestalozzistr. 11, Bretzfeld-Rappach 74626, Germany; E-Mail: Simone.Trefz@gmx.de

**Keywords:** chemical communication, aggregation pheromones, sexual pheromones, alarm pheromones, tritonymphs, house dust mite pest control

## Abstract

Despite the sanitary importance of the European house dust mite *Dermatophagoides pteronyssinus* (Trouessart, 1897), the pheromonal communication in this species has not been sufficiently studied. Headspace analysis using solid phase micro extraction (SPME) revealed that nerol, neryl formate, pentadecane, (6*Z*,9*Z*)-6,9-heptadecadiene, and (*Z*)-8-heptadecene are released by both sexes whereas neryl propionate was released by males only. Tritonymphs did not produce any detectable volatiles. In olfactometer experiments, pentadecane and neryl propionate were attractive to both sexes as well as to tritonymphs. (*Z*)-8-heptadecene was only attractive to male mites. Therefore it is discussed that pentadecane and neryl propionate are aggregation pheromones and (*Z*)-8-heptadecene is a sexual pheromone of the European house dust mite *D. pteronyssinus*. To study the potential use of pheromones in dust mite control, long-range olfactometer experiments were conducted showing that mites can be attracted to neryl propionate over distances of at least 50 cm. This indicates that mite pheromones might be useable to monitor the presence or absence of mites in the context of control strategies.

## 1. Introduction

Feces of the house dust mites *Dermatophagoides farinae* Huges 1961 and *D. pteronyssinus* (Trouessart, 1897) (Oribatida, Pyroglyphidae) are a major source for house dust allergens [1,2] and are responsible for 95% of all cases of house dust allergy [3]. Potential methods for mite control consist in airing and cleaning the bedclothes, structural measures to reduce suitable mite habitats, the use of fungicides to reduce fungi that serve as food, and acaricides to kill the mites [4]. One promising, potential control method that has not been studied sufficiently is the use of mite pheromones to manipulate their behavior. A large number of studies on mite pheromones revealed that these arthropods rely strongly on pheromonal communication to form aggregations, escape from enemies, find mates, and stimulate mating behavior in the other sex [5]. Therefore, pheromones could be used in traps to attract mites, either for monitoring, or in the context of a lure and kill strategy [6].

Pheromone studies have been performed on both *Dermatophagoides* species. This resulted in the identification of 2-hydroxy-6-methylbenzaldehyde as sexual pheromone for the American house dust mite *D. farinae* [7], neryl formate as aggregation pheromone for both species [8], and citral as putative defensive compound for *D. farinae* [9], which does also occur in *D. pteronyssinus* [10]. However, several compounds were described to be released by both species for which no biological function has been identified so far [5,10,11]. In addition, nothing is known on potential sexual pheromones in *D. pteronyssinus* and it is unclear which substances are released by which sex. Furthermore, studies with house dust mites so far only addressed pheromone release and reaction to pheromones in adult mites while the role of pheromones for nymphs remained unclear. The characteristic opisthosomal glands (“oil glands”), which are made responsible for the release of most pheromones in astigmatid mites [5] are present in nymphs as well [12] and they have also been reported to release pheromones [13,14,15].

To address the abovementioned open questions in the European house dust mite *D. pteronyssinus*, we (1) analyzed the headspace of mite cultures and the stage- and sex-specific release of volatile chemicals. Headspace analysis was used in contrast to mite extracts, because our aim was to study actual pheromonal communication i.e. the compounds that are released by the mites under “normal”, undisturbed conditions. Therefore, care was also taken not to disturb the mites during this analysis. In addition, we tested the response towards identified compounds (2) and studied the feasibility of using attractive pheromonal compounds in the context of control of house dust mites (3).

## 2. Experimental

### 2.1. Cultures

A stock culture of *D. pteronyssinus* was obtained from the company Insect Services GmbH, Berlin. Mites were kept on a diet of dried yeast, fish food (Tetramin) and wheat flour (2:1:2) in closed desiccators (Ø 150 mm, height 150 mm) at 70%–75% relative humidity and 22–24 °C. Mite species, sexes and developmental stages were identified using the keys provided by Hughes [16].

### 2.2. Chemical Analyses

Previous analysis of mite cultures by us has shown that mite extracts might miss important pheromonal components because pheromone storage in mite glands might be limited [17]. In addition, extracts can contain substances that are not normally emitted by mites, e.g., alarm pheromones or defensive compounds, which are released only at disturbance. Therefore, to study the sex specific release of chemical substances by undisturbed mites, headspace analysis using solid phase micro extraction (SPME) using undisturbed mites was performed. 100 adult females or males were placed in a 4 mL glass vial (Ø 13 mm, height 46 mm) together with 250 mg rearing substrate. The neck of the vial was covered with Teflon^®^ to prevent mites from escaping. To avoid disturbance of the mites, which can be detected by the presence of citral in the headspace, vials were closed with a screw cap with gauze (60 × 60 per cm^2^) after transfer of the mites and kept for 3 days at rearing conditions. After 3 days the gauze was carefully replaced by a rubber septum in order to not disturb the mites. An SPME-fiber (Stable Flex ^TM^ 65 μm PDMS-DVB coating) was introduced through the rubber septum in the headspace of the vial for 45 min. Afterwards, the SPME-fiber was introduced into the injector of a GC/MS system (Agilent: GC 6890N; MS: MSD 5973) and desorbed for 2 min at 300 °C. The gas chromatograph was equipped with a 30 m fused-silica-capillary HP5 column (Agilent) and programmed as follows: 60 °C for 3 min, then with 3 °C/min to 300 °C. All compounds were identified by comparison of mass spectra and gas chromatographic retention times to those of synthetic reference material. To verify that compounds were released by the mites and not by the culture medium, the headspace of pure medium was analyzed accordingly. For tentative quantifications of compounds selected for bioassays (see below), 10 to 30 mites were placed in 4 mL glass vials (Ø 13 mm, 46 mm high) and the volatiles in the headspace were analyzed by introducing an SPME-fiber for 30 min as described above. To determine the quantities released within these 30 min, peak areas from these analyses were compared with peak areas from calibration curves. These were obtained by SPME-analyses of the headspace of 4 mL glass vials containing filter papers with different concentrations of the respective synthetic compounds.

### 2.3. Synthesis

**Figure 1:**
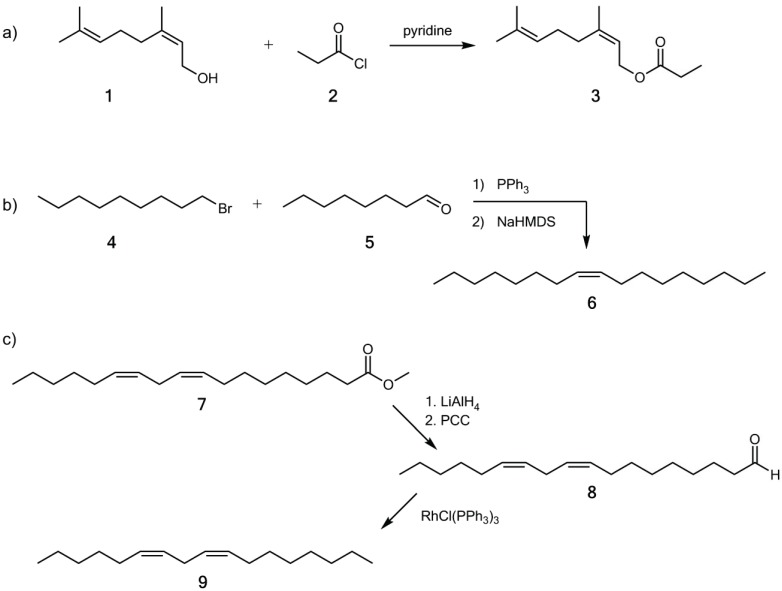
(**a**) Synthesis of neryl propionate (**3**); (**b**) Synthesis of (*Z*)-8-heptadecene (**6**); (**c**) Synthesis of (6*Z*,9*Z*)-6,9-heptadecadiene (**9**).

Neryl propionate (**3**) was synthesized from nerol (**1**) and propionyl chloride (**2**) (Figure 1). To an ice-cooled solution of nerol (1.54 g, 10 mM) in abs. pyridine (20 mL) propionyl chloride (0.93 g, 10 mM) was added slowly. After stirring over night at 0 °C the mixture was poured on ice, extracted with diethyl ether (3×), washed with HCl sol. (1 N) and sat. NaHCO_3_ sol., drying with Na_2_SO_4_ the solvent was removed, and the product purified by column chromatography on silica (hexane/diethyl ether 5:1. A chemically pure colorless oil was obtained (1.51 g, 7.2 mM, 72% yield). The *Z*/*E* ratio of the product was 97:3.

(*Z*)-8-Heptadecene (**6**) was synthesized by a Wittig reaction from 1-bromononane (**4**) and octanal (**5**) using the conditions of Pempo *et al.* [18]. Instead of *n*-butyllithium sodium hexamethyldisilazide (NaHMDS) was used as non-nucleophilic base. The product was purified by column chromatography on silica (hexane) and was obtained chemically pure in 72% yield. The *Z*/*E* ratio was 96:4. The spectroscopical data were identical to those already published [19].

(6*Z*,9*Z*)-6,9-Heptadecadiene (**9**) was synthesized from methyl linoleate (**7**). Reduction with lithium aluminium hydride furnished the corresponding alcohol [20] that was oxidized with pyridinium chlorochromate to yield (9*Z*,12*Z*)-9,12-octadecadienal (**8**) [21]. Decarbonylation of the latter using Wilkinson catalyst (RhCl(PPh_3_)_3_) [22,23] gave the desired hydrocarbon **9** in 88% yield in the last step after chromatography on silica (hexane). The product was diastereomerically pure. NMR data of the synthesized compound were identical to those published earlier [24].

### 2.4. Reaction to Synthetic Compounds in the Olfactometer

**Figure 2:**
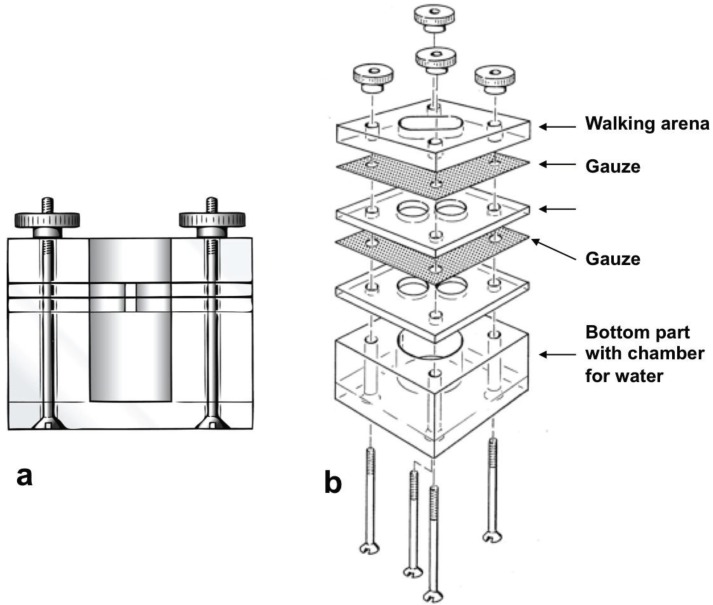
Static two-chamber olfactometer. (**a**) Lateral view. (**b**) Exploded drawing, showing the different components.

To test the behavioral activity of identified compounds, experiments were performed in a static two-chamber olfactometer (5 × 5 × 5 cm; Figure 2). It consists of one base element containing a perpendicular hole for water to increase humidity during the experiments. On top of the base element several perspex plates are placed containing two chambers for odor samples and a walking arena on top of the chambers. The chambers for odor samples are closed from below and from above by gauze with thread count of 60 × 60/cm^2^ for adult mites and 100 × 100/cm^2^ for nymphs. To prevent mites from climbing up the walls and to get into contact with the gauze of the walking arena, the walls of the chambers were coated with Teflon^®^. Single, well-fed mites were released on the walking arena and their allocation time in the walking arena above test and control chamber was registered for 600 s using [25] and compared statistically with Wilcoxon-matched-pairs-test for dependent data using [26]. Replications were discarded when mites were falling on their back, stuck to the Teflon^®^, escaped from the olfactometer, climbed up the wall or did not move during the experiment. Experiments were performed at 23 to 25 °C.

Filter paper discs (Ø 5 mm) were placed in the test chamber and the control chamber of the olfactometer and treated with synthetics dissolved in hexane (test) or with hexane (control), respectively. The tested quantities were equivalent to the estimated amounts released by 10 to 30 mites (see above). The following compounds were tested: 30 ng (*Z*)-8-heptadecene (30 μL of 1 ng/μL); 30 ng pentadecane (30 μL of 1 ng/μL); 2 μg (6*Z*,9*Z*)-6,9-heptadecadiene (20 μL of 0.1 μg/μL); 0.1 ng neryl propionate (10 μL of 0.01 ng/μL). A recent study with extracts of mite oil glands revealed that most of the compounds evaporate within the first 600 s after application on filter paper [27]. Therefore, it is very likely that the test mites were exposed to the complete amount of the tested quantities.

### 2.5. Use of Neryl Propionate as Bait

**Figure 3:**
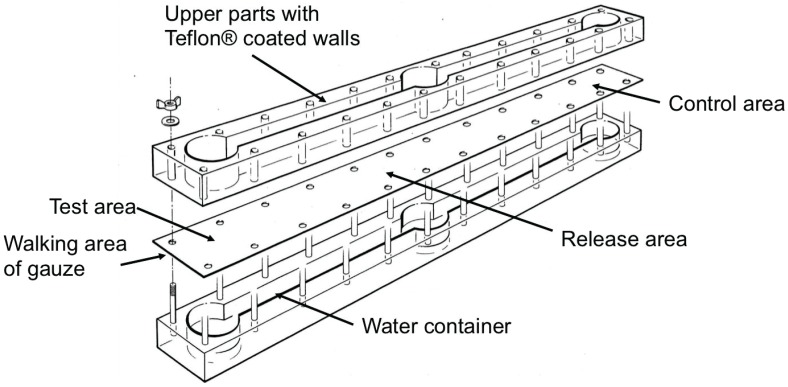
Long range olfactometer used to test the attraction for house dust mites *D. pteronyssinus* of chemical substances over larger distances.

To test the ability of neryl propionate to attract mites over larger distances, a long range olfactometer was used (1100 mm × 100 mm × 80 mm; Figure 3). It consists of one circular release area in the center as well as one circular test and control area at each end. The bottom part contains a water basin to maintain humidity covered by a walking arena of gauze (60 × 60/cm^2^). The walls of the upper part were coated with Teflon® to prevent mites from escaping. A Petri dish (Ø 40 mm) containing paraffin oil was placed on the gauze in the test area and the control area. 2.5 μg Neryl propionate dissolved in hexane (25 μL of 0.1 μg/μL) was added to the Petri dish in the test chamber and 25 μL hexane to the Petri dish in the control chamber. Thus, the paraffin oil served both, as trap for the mites and to ensure a slow release of the neryl propionate. For each replicate, 100 mg substrate from the mite cultures was placed on a filter paper (Ø 60 mm) in the center of the release area. The number of mites in the vials in the test and the control area were counted after 48 h.

## 3. Results

### 3.1. Chemical Analyses

#### 3.1.1. Headspace of Mite Cultures

The analysis showed the presence of pentadecane, heptadecene, and heptadecadiene in the colony. Derivatisation with dimethyl disulfide (DMDS) showed that the unsaturated hydrocarbons were (*Z*)-8-heptadecene and (6*Z*,9*Z*)-6,9-heptadecadiene, previously identified in total extracts of *D. pteronyssinus* [10]. A minor component of the headspace exhibited a mass spectrum similar to neryl acetate, but showing an ion at *m/z* 57 instead of *m/z* 43. Therefore this compound was proposed to be neryl propionate, which proved to be correct after comparison with the synthesized compound. In addition, nerol and neryl formate were identified.

#### 3.1.2. Sex Specific Release of Compounds

SPME-analyses of headspace from cultures containing only males, only females, or only tritonymphs revealed that both sexes release nerol, neryl formate, pentadecane, (6*Z*,9*Z*)-6,9-heptadecadiene, and (*Z*)-8-heptadecene. In contrast, neryl propionate was only found in the headspace of cultures of male mites. No compounds were found in the headspace of tritonymphs.

### 3.2. Attraction to Synthetic Compounds

**Figure 4:**
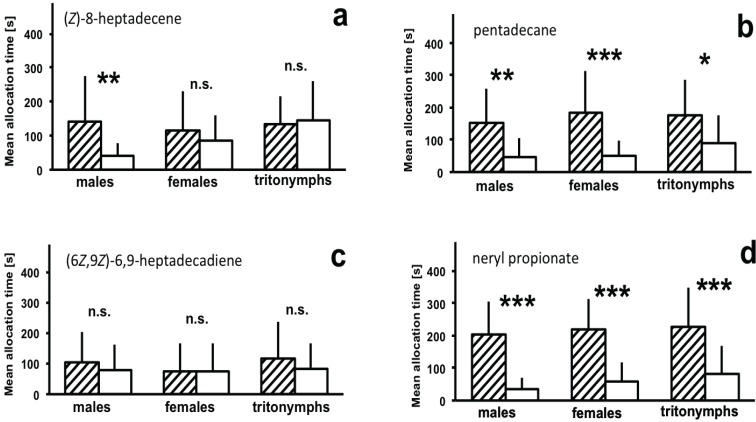
Reaction of males, females and tritonymphs of *D. pteronyssinus* to synthetic odors. The graph presents the mean allocation time (+S.D., n = 25) in the odor field of a two-chamber olfactometer above chambers with a filter paper containing a synthetic odor dissolved in hexane (hatched bars) or control chambers containing a filter paper with the respective amount of hexane (white bars). n.s.—no significant difference between test and control field; * *p* < 0.05; ** *p* < 0.01; *** *p* < 0.01 (Wilcoxon-matched pairs test) (**a**) 30 ng (*Z*)-8-heptadecene (30 μL of 1 ng/μL) (**b**) 30 ng pentadecane (30 μL of 1 ng/μL) (**c**) 2 μg (6*Z*,9*Z*)-6,9-heptadecadiene (20 μL of 0.1 μg/μL) (**d**) 0.1 ng neryl propionate (10 μL of 0.01 ng/μL).

Whereas (6*Z*,9*Z*)-6,9-heptadecadiene did not cause any reaction by the test mites, (*Z*)-8-heptadecene was attractive for male mites (Figure 4). Mites of both sexes were attracted to pentadecane and neryl propionate.

### 3.3. Potential Use of Neryl Propionate as Bait for House Dust Mite Control

**Figure 5:**
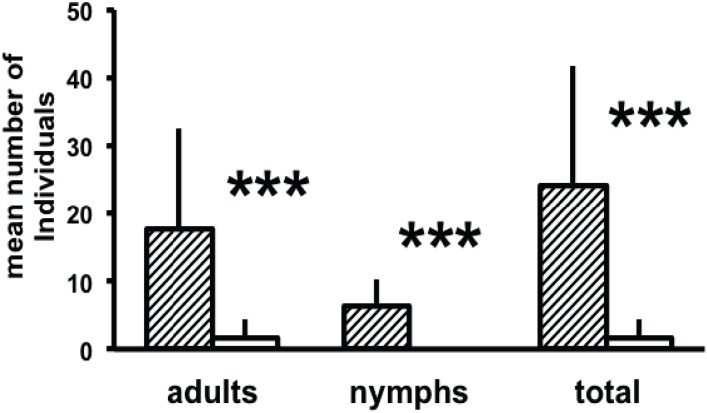
Mean number of adults, nymphs, and total (+S.D., n = 9) of *D. pteronyssinus* in test (shaded bars) and control Petri dishes (white bars) of a long range olfactometer after 48 h. The test Petri dish was baited with neryl propionate. *** *p* < 0.001 (Whitney-Mann U-test).

After 48 h, significantly more adults and nymphs were found in the Petri dish baited with neryl propionate as compared to the control (Figure 5). This demonstrates that mites can be lured over distances of at least 50 mm by attractive pheromonal compounds.

## 4. Discussion

Our chemical analyses revealed the presence of nerol, neryl formate, pentadecane, (6*Z*,9*Z*)-6,9-heptadecadiene, and (*Z*)-8-heptadecene in the headspace of both sexes of the European house dust mite *D. pteronyssinus* and neryl propionate in the headspace of males only. Because the studied mites were undisturbed, we are confident that these compounds are not related to alarm communication or defense. From these compounds, neryl formate, (6*Z*,9*Z*)-6,9-heptadecadiene, and (*Z*)-8-heptadecene were already identified in earlier studies [8,10] but no function was suggested for the two hydrocarbons. Their similar structure, obvious from the fact that (*Z*)-8-heptadecene can also be termed (*Z*)-9-heptadecene, indicates a common biosynthetic origin. Nerol and neryl propionate have not been found in the headspace of *D. pteronyssinus* so far. Interestingly, no volatiles were found in the headspace of tritonymphs, supporting the idea that the release of pheromones in *D. pteronyssinus* is stage specific, as has been reported also for other mite species [13,14].

In our studies on the biological activity, pentadecane and neryl propionate were shown to be attractive to males, females, and tritonymphs, (*Z*)-8-heptadecene was attractive only to males and the other compounds caused no reaction. Thus, pentadecane and neryl propionate match the definition of aggregation pheromones, which cause aggregative behavior in conspecifics of both sexes or in the same sex as the emitter [28]. In fact, house dust mites have been reported to form aggregations [29]. Generally, aggregations of arthropods are considered to enhance mate finding, to facilitate feeding, to improve survival under adverse environmental conditions and to provide protection from enemies [6,28]. Due to the fact that tritonymphs reacted to both substances, a function of aggregations in the context of mate finding seems unlikely for *D. pteronyssinus*. The idea that mite feeding is improved by living in aggregations can also be excluded. House dust mites feed on pollen, spores of microorganisms, fungal mycelia, bacteria, plant fibers, lepidopterous scales, animal dander, and skin scales of birds [30]. It is very unlikely that consumption on these food sources is density independent. Rather, aggregations could protect mites from desiccation [31] because water loss reduction was demonstrated in aggregations for the American house dust mite [32]. Likewise, aggregations might provide protection by the “safety in numbers” strategy against the natural enemies of the mites, e.g., predatory mites, silverfish, dust lice, or pseudoscorpiones [30]. In addition, the pheromone might also serve several functions at the same time, e.g., to provide protection against desiccation and against natural enemies.

The fact that only males but not females reacted to (*Z*)-8-heptadecene strongly indicates a function as sexual pheromone for this compound. Surprisingly, however, this compound is released not only by females, but also by males. One explanation for this apparent contradiction could be provided by studies in other animal species, likewise showing the release of female sexual pheromones in young males. In the rove beetle *Aleochara curtula*, this is interpreted as an attempt by young males to avoid aggressions by older males [33]. In the parasitoid wasp *Lariophagus distinguendus*, young males are discussed to release female pheromones to distract older males to obtain more matings [34]. Possibly, the release of (*Z*)-8-heptadecene by males serves a similar function in *D. pteronyssinus*.

Thus, the present study identified pentadecane and neryl propionate as two new aggregation pheromone components for *D. pteronyssinus,* (*Z*)-8-heptadecene as one potential new sexual pheromone component, as well as nerol for which no function could be described. This adds to the findings of earlier studies by Kuwahara *et al.* [10] and Sato *et al.* [11] who identified a number of compounds for *D. pteronyssinus*, as well as to the recent work by Skelton *et al.* [8] describing neryl formate as aggregation pheromone of this species (Table 1).

**Table 1:** Chemical compounds released by the house dust mite *D. pteronyssinus* and their proposed pheromonal function as reported in the literature and discussed in the present study.

Compound	Demonstration of its presence	Suggested function
Neral	[10]	unknown
Geranial	[10]	unknown
Rhizoglyphinyl formate (2-Formyl-3-hydroxybenzyl formate)	[11]	unknown
Nerol	this study	unknown
Neryl formate	[8], this study	aggregation pheromone [8]
Neryl propionate	this study	aggregation pheromone, this study
Pentadecane	[10], this study	aggregation pheromone, this study
Geranyl formate	[10]	unknown
3-Hydroxybenzene-1,2 -dicarbaldehyde	[5]	unknown
Heptadecane	[5]	unknown
(*Z*)-6-Pentadecene	[5]	unknown
(6*Z*,9*Z*)-6,9-Heptadecadiene	[10], this study	unknown
(*Z*)-8-Heptadecene	[10], this study	female sex pheromone, this study

To study the potential use of pheromones to control house dust mites, the ability of pheromones to attract mites over larger distances was examined using our newly identified male released aggregation pheromone neryl propionate, which is attractive to both sexes as well as to tritonymphs of *D. pteronyssinus.* The study demonstrates that adult mites and nymphs can be attracted to neryl propionate over at least 50 cm. The 100 mg medium that was placed in the olfactometer to release the mites contained many hundred of individuals whereas only a mean number of about 24 mites were detected in the trap after 24 h. Therefore only a small fraction of released mites could be retrieved in the trap. This indicates that neryl propionate is not suitable for a lure and kill strategy [6] to directly control the mites. Rather, it might be possible to use this compound for monitoring, e.g., to verify the presence or absence of mites in a bedroom before and after control measures, respectively. This strategy is commonly used in integrated pest management (IPM) of agricultural pest insects [35]. For this strategy to work, only a small fraction of mites has to be caught and it is not required that mites are attracted from the whole area of infestation. More studies are required to answer the question, if other pheromonal compounds could also be used in mite control.

## 5. Conclusions

We demonstrate that males and females, but not tritonymphs of the house dust mite *D. pteronyssinus* release pheromonal signals. We identified pentadecane and neryl propionate as two new aggregations pheromones, which are attractive to both sexes and to tritonymphs of the house dust mite *D. pteronyssinus*, as well as (*Z*)-8-heptadecene as potential sexual pheromone, which is attractive to males only. In order to use pheromones in control strategies of house dust mites, we demonstrate that neryl propionate could possibly be used to attract mites over larger distances, e.g., to monitor the presence or absence of house dust mites.
